# Why Measure Patient Experience in Physical Therapy?

**DOI:** 10.1186/s40945-021-00105-2

**Published:** 2021-05-03

**Authors:** Jacob Eversole, Ashton Grimm, Nikita Patel, Kelly John, Alessandra N. Garcia

**Affiliations:** College of Pharmacy & Health Sciences, Doctor of Physical Therapy Program, Campbell University, 4150 US 421 South, Lillington, NC 27546 USA

**Keywords:** patient experience, patient reported experience measures, physical therapy

## Abstract

**Background:**

Patient experience is an important component of quality and patient centered health care not fully explored in physical therapy.

**Main body:**

This article addresses (1) concept of patient experience, (2) importance of capturing the patient experience, (3) measures to capture patient experience and whether these measures exhibit psychometrically sound measurement properties, (4) relationship between patient experience and clinical effectiveness outcomes, and (5) clinical applications of patient experience measures in the outpatient physical therapy setting, including suggestions for future studies.

**Short conclusion:**

Employing patient experience measures into physical therapy practice may be an important key to improve clinical effectiveness outcomes and provide excellent patient-centered care delivery. An area of continued research should be focused on demonstrating the generalizability and measurement properties of patient reported experience measures for the musculoskeletal outpatient physical therapy population focusing first on the most common musculoskeletal conditions such as cervical, low back, and shoulder pain.

Improving quality of care is an important focus for physical therapists. Quality of care can be evaluated based on inter-related domains of clinical effectiveness, safety, and patient experience. These related domains allow for improvements to be made in one area while addressing another. For example, addressing areas having been determined to need improvement through the information provided by a measure of patient experience could also potentially result in improvements to either of the two domains [1]. While a variety of patient experience measures have been developed for medical practice, including specific questionnaires for specialty practices such as rheumatology, pediatrics, respiratory medicine, and cardiology [2], literature on the utilization of patient experience measures for musculoskeletal disorders in outpatient physical therapy setting is not well and fully explored.

Our view is that employing patient experience measures into physical therapy practice to objectively account for the patients’ perceptions of their health and experiences across various components is an important key to improve clinical effectiveness outcomes and provide excellent patient-centered care delivery. In this article, we discuss the (1) concept of patient experience, (2) importance of capturing the patient experience, (3) measures to capture patient experience and whether these measures exhibit psychometrically sound measurement properties, (4) relationship between patient experience and clinical effectiveness outcomes, and (5) clinical applications of patient experience measures in the outpatient physical therapy setting, including suggestions for future studies.

## Defining patient experience

Patient experience involves the sum of all interactions that patients have with the healthcare system, including their care from health plans, healthcare providers, and staff in inpatient and outpatient settings. It is shaped by an organization’s culture, that influence patient perceptions across the continuum of care [3]. At its core, patient experience can be defined as any feedback given from the patient following a clinical encounter about their perceptions of met needs [4]. It is important to note that patient experience is influenced by different factors, dependent on what setting a patient is in. Items as continuity & physical comfort on measures of patient experience in inpatient settings is one such example [6]. Patient experience can be compiled into relational pillars (e.g., interpersonal aspects of the quality of care received such as communication, respect and dignity, and emotional support); and functional pillars (e.g., environmental factors such as facility characteristics, type of service being provided as well as patient characteristics, such as sociodemographic characteristics, clinical history, prior health care-seeking behavior) [4, 5, 7]. It is worth noting that, while the terms patient experience and patient satisfaction are often used interchangeably, they are not the same construct. Patient satisfaction combines patient experience with their health outcomes and confidence in their providers and the healthcare system to indicate whether their needs and expectations have been met [3, 7]. Patients’ expectations can be shaped by their place within society and their community and family context [5]. Patient satisfaction has been criticized for these inherent sources of bias [6]. In fact, patients often express higher levels of satisfaction due to gratitude bias and other factors [2], which leads to an optimistic picture of performance [6]. Therefore, it is important to differentiate between how people feel about things (satisfaction), and what happened during care and the extent to which patient’s needs were met (experience) [6]. That said, while patient experience can certainly influence patient satisfaction, they are distinct concepts that should be treated and measured as such [2, 6].

## Why is it important to capture patient experience?

The overall quality of healthcare experience is intimately tied to the patient’s experience [7]. While improving patient experience has an inherent value to patients, it may also be associated with clinical processes and outcomes [8]. One bases their evaluation of rehabilitation service quality on a number of heterogeneous details across differing components involved in their experience with one such focus being their individual physical therapist. Some of the variables related to the physical therapist involved in their care includes items such as their perspective of physical therapists’ interpersonal manners, willingness to provide information and education, and technical expertise [9]. In a similar manner, there are variables involved which are directed more towards the field of physical therapy and healthcare practice rather than the physical therapist as an individual. Examples of such variables include but are not limited to expectations and perspectives towards the profession, the clinic, and their preconceptions on their potential recovery and the ability for physical therapy to address their condition. Capturing this information can help identify either the components of care that are performed well, or the parts that need improvement based on what the patient’s value most highly [6, 9]. Furthermore, better adherence to treatment plans and medical advice in addition to subsequent improvements in health outcomes can occur with improvements in patients’ experiences during their time in a healthcare environment [9]. Because of this, it is not enough to uniquely rely on clinical outcomes as determinants of a successful health care experience. With this relationship between experience and clinical outcomes, addressing the components of care that were identified as needing improvement through an objective experience measure can subsequently improve not only the patient’s experience, but also their scores on objective outcome measures [4, 7, 9]. To know what does not work for patients can help breed creativity for new ways of care. To know what does work can help create security and consistency. In our view, both are crucial results to obtain from objective measures. Evidence suggests that interpersonal skills (namely provider and staff communication) and logistics of healthcare delivery, including items such as safety, efficacy can be identified through measures of patient experience as areas needing improvement to increase scores on patient outcomes [3, 4, 9].

## How can we measure patient experience?

Measuring patient experience is unique from measuring patient outcomes and can be done with patient reported experience measures (PREMs). Unlike the commonly known patient reported outcome measure (PROMs), PREMs look at the patients experience with the healthcare system while receiving care, and objectively measure their experiences rather than simply subjective reports of satisfaction or outcomes [2]. While PROMs are standardized and validated questionnaires that are given to patients to evaluate how clinically effective an intervention has been through the patients’ perspective [2]. Both PREMs and PROMs can be used to measure the quality of care and help guide healthcare services [2].

PREMs are questionnaires or surveys that can be used as a marker of healthcare quality based on the patient's perspective [2]. PREMs can also serve as a means of determining areas for growth within the healthcare system [9]. Two classifications of PREMs exist with the functional classification examining the objective components such as facilities whereas relational PREMs assess a patient’s experience based on the relationships they had with providers during their treatment (Table 1) [2]. Developing PREMs that are validated with appropriate measurement properties has been challenging.

**Table 1:** Examples of functional and relational aspects assessed by PREMs

Functional	• Waiting times in the sequence of treatment [6] • Patient safety [6] • Physical environment [10] • Medicine availability [10] • Medical information [10] • Staff behavior [10] • Doctor behavior [10] • Hospital infrastructure [11] • Information on tests [12] • Prompt access [12] • Handling patient feedback [12] • Quality of care and overall care of health professionals [12]	• Environment and facilities [12] • Information provision [13] • Effective treatment [13] • Timely, tailored, and expert management of physical symptoms [13] • Coordinated care between settings [13] • Clean, safe, comfortable environment [7] • Clinic access [14] • Hospital standards [14] • Continuity and transition [15] • Rehabilitation care and organization [16] • Registration Process [17]
Relational	• Sensitivity to patients’ changes [6] • Emotional support [6] • Duration of [physical therapist] attendance [6] • Interruptions during delivery of care [6] • Providing information and education [6] • Nurse communication [10] • Doctor communication [10] • Pain management [10] • Medication and symptom communication [10] • Whether the doctors were understandable [12] • Doctors’ professional skills [12] • Nursing care [4] • Whether doctors and nurses were interested in the patient’s problems [12] • Physical and emotional needs [13] • Respect and privacy [13] • Coordination of care [13]	• Care and involvement in decision making [13] • Emotional and psychological support [7] • Involvement of family and caregivers in decisions [7] • Kindness [7] • Dignity [7] • Compassion [7] • Transparency [7] • Honesty [7] • Disclosure [7] • Clear communication [7] • Respect [7] • Relief of fear and anxiety [7] • Pre-visit communication [14] • Social environment [16] • Patient-therapist interaction [17] • Courtesy of receptionist [17] • Personalized Therapy [18]

### Examples of PREMs

There are a number of PREMs available for use in both the inpatient and outpatient settings and for specific medical conditions. The decision of which one to use in clinical practice should take the questionnaires’, measurement properties into consideration. Table 2 displays a list of some PREMs that are primarily used for patients with musculoskeletal disorders who received outpatient physical therapy. We endeavored to follow the criteria for good measurement properties recommended by the COnsensus-based Standards for the selection of health Measurement Instruments (COSMIN) to report the measurement properties of the PREMs [19].

**Table 2:** Examples of PREMs for musculoskeletal disorders in outpatient physical therapy

PREM	Population	Patient experience aspects	Measurement properties	Strengths	Limitations	Reference
PEPAP-Q: Patient Experiences in Postacute Outpatient Physical Therapy Settings	Patients participating in rehabilitation centers for MSK conditions MSK disorders: surgical recovery from lower back injury, upper limb fracture, lower limb fracture, shoulder injury, and knee injury	Professionals' attitudes and behavior (providing information and education, sensitivity to patients' changes, and emotional support) and 4 factors that conceptually reflect organizational environment (duration of attendance, interruptions during care delivery, waiting times, and patient safety).	PREM development and content validity Internal Consistency: + Reliability (ICC): + Hypothesis testing for construct validity: +	The PEPAP-Q can be considered more effective than generic that does not reflect what truly matters to a patient in a specific context.	This PREM was developed in Spanish, and the English translation has not been revalidated Questionnaire is limited to the outpatient setting assessing and treating MSK related conditions Does not assess technical aspects of care (i.e. PT’s level of education)	Medina-Mirapeix F, del Baño-Aledo ME, Martínez-Payá JJ, Lillo-Navarro MC, Escolar-Reina P. Development and validity of the questionnaire of patients’ experiences in postacute outpatient physical therapy settings. *Phys Ther.* 2015;95(5):11.
Picker MSD questionnaire	Patients who received outpatient care from a spine clinic MSK disorders: back pain and neck pain	Access to care (six items), information and education (six items), respect for patients’ preferences (five items), emotional support (four items), coordination of care (five items), continuity and transition (four items), overall impression (four items)	PREM development Internal Consistency: + Hypothesis testing for construct validity +	Questions were developed from interviews with health care provides (2 physicians, 2 PTs, 1 chiropractor, and 1 osteopath) and interviews and focus groups with patients	This PREM can be used in any healthcare setting that addresses musculoskeletal disorders; therefore, it does not apply only to Physical Therapy Services	Jenkinson C, Coulter A, Gyll R, Lindstrom P, Avner L, Hoglund E. Measuring the experiences of health care for patients with musculoskeletal disorders (MSD): development of the Picker MSD questionnaire. Scand J Caring Sci. 2002;16(3):329-333.
Re-PEQ: Rehabilitation Patient Experiences Questionnaire	Patients who received rehabilitation for rheumatological disorders MSK disorders: rheumatoid arthritis, ankylosing arthritis, osteoarthritis, and other rheumatic diagnoses	Rehabilitation care and organization, information and communication, availability of staff, and social environment	PREM development and content validity Internal Consistency: + Hypothesis testing for construct validity: +	Only known PREM for patients with rheumatic conditions who received rehabilitation Collected data from both outpatient and inpatient rehabilitation centers; therefore, data is not solely applicable to outpatient setting	Only assess after receiving treatment and therefore cannot provide data about sensitivity to change or responsiveness (i.e. MDC or MCID)	Grotle M, Garratt A, Lochting I, et al. Development of the rehabilitation patient experiences questionnaire: data quality, reliability and validity in patients with rheumatic diseases. J Rehabil Med. 2009;41(7):6.
MedRisk Instrument for Measuring Patient Satisfaction^a^ With Physical Therapy Care (MRPS)	Patients who were receiving outpatient physical therapy for one or more musculoskeletal conditions MSK disorders: Pain at one or more of the following locations: cervical spine, lumbar and thoracic spine, wrist and hand, upper extremity, or lower extremity	Patient- therapist interaction (communication and respect), non patient-therapist interaction (registration process and courtesy of receptionist), global measures of satisfaction	PREM development and content validity Internal Consistency: + SEM: ?	Completed a follow-up study that assessed discriminant and concurrent validity	Only applicable for patients who are covered by workers' compensation; further data is needed for patients who are receiving physical therapy without said coverage	Beattie P. Patient Satisfaction With Outpatient Physical Therapy: Instrument Validation. *Physical therapy*. 2002;82(6):557-565. doi:10.1093/ptj/82.6.557 Beattie P, Turner C, Dowda M, Michener L, Nelson R. The MedRisk Instrument for Measuring Patient Satisfaction With Physical Therapy Care: a psychometric analysis. *The journal of orthopaedic and sports physical therapy*. 2005;35(1):24-32. doi:10.2519/jospt.2005.35.1.24

+ (sufficient), - (insufficient),? (indeterminate); ^a^states satisfaction but measures patient experience; *MSK* musculoskeletal, *SEM* Standard Error of Measurement

## PREM vs clinical effectiveness outcomes

The importance of the utilization of PREMs in relation to clinical outcomes is evident by the correlation between these two measures, with one example of such instance demonstrated through the improvement in outcome scores for individuals undergoing hip, knee, and hernia repairs [2, 4, 6, 8]. Of additional importance is evidence suggesting that satisfaction and quality of service is not indicative of patient experience [9]. Some findings contradicting the positive association between experience and outcomes exist, specifically in scenarios where communication was with physicians only or when experience measures were taken later than one month after the patient interaction of interest [1]. Experience measures provide insight on the place of treatment and take into account the facilities and various team members involved instead of only the primary provider [2, 9]. While clinical outcomes are an important component of health care and serve as a measure of a successful bout of treatment, experience measures are also critical in this analysis as evident by this information in conjunction with the previously mentioned clinical benefits. Physical therapists face numerous challenges in everyday practice and are constantly analyzing information during patient management so the addition of information from PREMs could further increase this complexity if applied in our decision making with respect to intervention choice. To avoid this, the insight gained from these experience measures should not be focused on clinical reasoning, but instead can serve as guidance with respect to areas of professional commitment that may require more attention than would otherwise be considered and help clarify areas of importance to patients that may have been unknowingly neglected.

## Clinical applicability and suggestions for future studies

A few common relational aspects of patient experience that presented in past studies were patient-therapist interactions and interpersonal skills [2, 4]. Some other common relational aspects of care identified were emotional support, sensitivity to patients’ changes, and information and education [6]. Within functional aspects of patient experience, a few other values presented, including communication between healthcare settings and the technical skills of their therapist [4]. Patients also valued the brevity of the registration process, waiting times in the sequence of treatment and a clean, safe and comfortable environment [2, 6, 7].

By making it a point to address aspects of patient experience that are valued by the patient, we may improve the overall patient experience, the quality of care and patient outcomes, but measuring these values in a validated questionnaire has been a challenge in an outpatient physical therapy world. Some different PREMs have been developed that each have their own strengths and weaknesses (Table 2). The Picker MSD and PEPAP-Q each have a wide array of relational and functional aspects of patient experience. For example, the Picker MSD has strong and reliable measurement properties, but is not a PT specific questionnaire. Of the PREMs listed in Table 2, The PEPAP-Q would be the most complete to use in clinical practice for a variety of MSK conditions. This PREM encompasses the highest and most appropriate measurement properties with 3 relational and 4 functional aspects of patient experience. However, more studies need to be done to revalidate the English version. Once we are better able to measure the patient experience and understand who is best to measure it, more improvements can be made in the clinic to improve the quality of healthcare, not only in doctors’ offices, but outpatient physical therapy clinics as well. An area of continued research should be focused on demonstrating the generalizability and measurement properties of PREMs for the musculoskeletal outpatient physical therapy population focusing first on the most common musculoskeletal conditions such as cervical, low back, and shoulder pain. Furthermore, context related effects of the delivery of these measures should be further explored in order to optimize the patient experience in outpatient physical therapy settings.

## Data Availability

Not applicable.
